# *Cryptococcus neoformans* infection presenting as a mediastinal mass in an immunocompetent child with parrot exposure: a case report and literature review

**DOI:** 10.3389/fmed.2026.1771746

**Published:** 2026-02-23

**Authors:** Guangxian Yang, Siping He, Jinghua Wang, Sijing Yu, Shuju Zhang, Wenwen Fan

**Affiliations:** 1 Department of Cardiothoracic Surgery, The Affiliated Children's Hospital of Xiangya School of Medicine (Hunan Children's Hospital), Central South University, Changsha, Hunan, China; 2 Department of Radiology, The Affiliated Children's Hospital of Xiangya School of Medicine (Hunan Children's Hospital), Central South University, Changsha, Hunan, China; 3 Department of Infectious Diseases, The Affiliated Children's Hospital of Xiangya School of Medicine (Hunan Children's Hospital), Central South University, Changsha, Hunan, China; 4 Department of Medical Genetics, The Affiliated Children's Hospital of Xiangya School of Medicine (Hunan Children's Hospital), Central South University, Changsha, Hunan, China; 5 Clinical Nursing Teaching and Research Section, The Second Xiangya Hospital of Central South University, Changsha, Hunan, China

**Keywords:** *Cryptococcus neoformans*, mediastinal mass, parrot exposure, pediatric infection, targeted metagenomic next-generation sequencing

## Abstract

*Cryptococcus neoformans* typically causes pulmonary or central nervous system (CNS) infections, but mediastinal mass as its primary manifestation is rare—especially in immunocompetent children with pet parrot exposure. This study reports a 7-year-old girl who presented with recurrent fever and a mediastinal mass secondary to *Cryptococcus neoformans* infection, with a 5-month history of daily contact with parrot feces. Conventional diagnostic tests (e.g., fungal culture, serology) were negative, and the diagnosis was confirmed by targeted metagenomic next-generation sequencing (tNGS) of bronchoalveolar lavage fluid (BALF). The patient received a three-phase antifungal regimen: induction with amphotericin B + flucytosine, consolidation with fluconazole, and maintenance with low-dose fluconazole. After one year of treatment, the mediastinal mass nearly resolved, and no recurrence was observed. A literature review, supplemented with specific cases of parrot-associated *Cryptococcus neoformans* infection, highlights that parrot exposure is an underrecognized risk factor for pediatric cryptococcosis, and tNGS significantly improves diagnostic efficiency for atypical extrapulmonary manifestations. This case emphasizes the importance of inquiring about pet bird exposure in children with unexplained mediastinal masses and fever, and supports the use of tNGS for early, non-invasive diagnosis.

## Background

Most chest masses in children are located in the mediastinum and can arise from congenital anomalies, benign or malignant tumors, or infections, with neoplasms being the most common cause (1). The mediastinum is a relatively small anatomical space composed of tissues from diverse embryological origins. Tumors may originate from the thymus, mediastinal lymph nodes, or other soft tissues, and can show epithelial, hematopoietic, mesenchymal, or neurogenic differentiation. Common pediatric mediastinal tumors include lymphoma, neuroblastoma, thymoma, T-cell acute lymphoblastic leukemia (T-ALL), and germ cell tumors (2). The prevalence of these tumors varies significantly with age (3). Neoplasms such as lymphoma (the most common pediatric mediastinal tumor) and germ cell tumors often dominate clinical suspicion, while fungal infections are rarely considered.

*Cryptococcus neoformans* is an opportunistic fungus transmitted via inhalation of aerosolized spores, with environmental reservoirs primarily in nitrogen-rich substrates like bird droppings (4). Parrots (e.g., tiger-skin parrots, African gray parrots) are high-risk carriers, as their feces and feather dust contain high concentrations of viable spores. While pediatric cryptococcosis is historically linked to immunodeficiency (e.g., HIV, organ transplantation), recent data show 19% of cases occur in immunocompetent children (5). However, mediastinal involvement in these children is extremely rare and often misdiagnosed as a tumor (6).

This study presents a case of a 7-year-old immunocompetent child with a *C. neoformans*-induced mediastinal mass linked to parrot exposure, and systematically reviews 4 recent cases (2010–2024) to compare diagnostic strategies, treatment regimens, and outcomes, highlighting the value of tNGS in early diagnosis.

## Case presentation

A 7-year-old girl presented with a high fever and chills, who from an urban family in Yulin, Guangxi Province, China, was born at full term via vaginal delivery with a birth weight of 3.4 kg. Prior to the onset of illness, her physical growth and neurodevelopment were consistent with age-related milestones. She lived with her parents (non-consanguineous marriage) and had a 10-year-old elder brother. The fever occurred 3–4 times daily, recurring every 5–6 h, with a maximum temperature of 40 °C. Initially, she was treated at home for two days with oral Chinese medicine and ibuprofen, but showed no significant improvement. She was then admitted to a local district-level hospital, where peripheral blood tests revealed a white blood cell (WBC) count of 14.49 × 10^9^/L, neutrophil count of 11.12 × 10^9^/L, and neutrophil percentage of 76.7%. C-reactive protein (CRP) was elevated at 44.8 mg/L. Oseltamivir and antibiotics including cefaclor were administered but the fever persisted. She was then transferred to a municipal-level hospital for further evaluation. Repeat blood tests confirmed elevated neutrophil count and CRP. Erythrocyte sedimentation rate (ESR) was elevated at 45 mm/h. Cytokine testing showed elevated levels of IFN-*γ* (6.27 pg./mL; normal ≤ 4.43) and IL-6 (56.66 pg./mL; normal ≤ 11.09), and normal level of IL-2, IL-4, IL-10, TNF-*α*, and IL-17. Respiratory pathogen screening was negative for respiratory syncytial virus (RSV), adenovirus, *Legionella pneumophila*, parainfluenza virus, *chlamydia pneumoniae*, *mycoplasma pneumoniae*, and tuberculosis (T-SPOT test). Chest CT showed a lesion in the lower lobe of the right lung, consistent with infection, as well as enlarged right hilar and mediastinum with heterogeneous density. Our treatment included intravenous ampicillin-sulbactam for two days, followed by a combination therapy with azithromycin for five days. The patient’s fever initially subsided for three days but recurred on the fourth day, reaching 38.5 °C. A follow-up chest CT showed partial resolution of the right lower lobe lesion; however, the right hilar and mediastinal enlargement remained.

**Figure 1:**
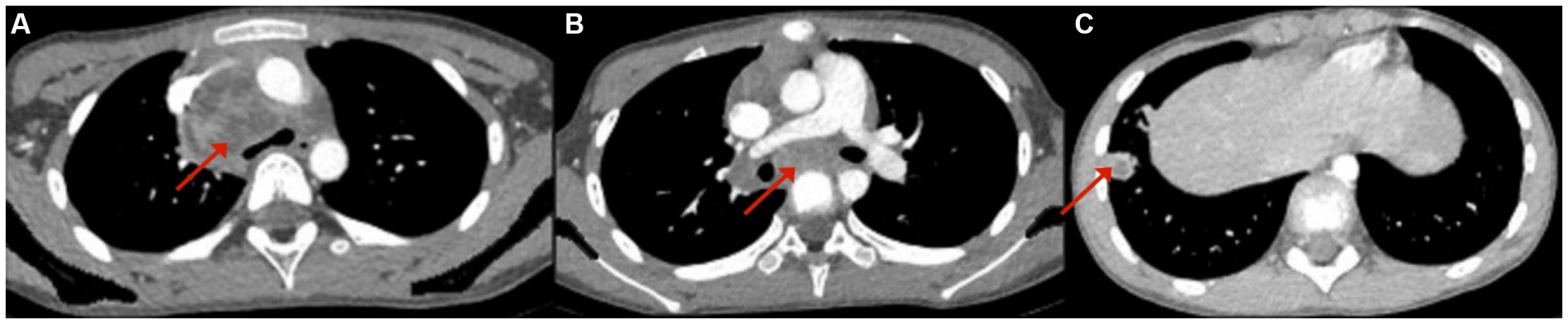
Chest CT: a patchy soft tissue density shadow with obvious uneven enhancement can be seen in the right anterior upper mediastinum, paratracheal vena cava, tracheal prominence, and right pulmonary hilum area, with patchy areas without enhancement visible inside **(A,B)**. The nodular high-density shadow in the anterior basal segment of the lower lobe of the right lung shows annular enhancement **(C)**.

After 16 days of on-and-off fever and a persistent mediastinal mass, the patient was transferred to our hospital for further evaluation. Peripheral blood testing revealed normal blood cell counts, a negative EBV-DNA fluorescence quantitative test, a CRP level of 9.11 mg/L (normal), and a slightly increased ESR at 27 mm/h. Autoimmune screening showed negative lupus serology. Tests for EBV, hepatitis C antibody, hepatitis B surface antigen (HBsAg), syphilis-specific antibody (Anti-TP), and HIV antibody (Anti-HIV) were all negative. Complement levels showed an elevated C3 at 1.47 g/L (reference range: 0.62–1.21 g/L), and immunoglobulin E (IgE) was increased to 214 IU/mL (reference <90 IU/mL). Other immunoglobulins, including IgM, IgG, IgA, and complement C4, were within normal limits. *Mycoplasma pneumoniae* antibody titers were positive at 1:40, positive at 1:80, and weakly positive at 1:160. Tumor marker screening revealed normal levels of alpha-fetoprotein (AFP), carcinoembryonic antigen (CEA), *β*-human chorionic gonadotropin (β-HCG), and neuron-specific enolase (NSE). Throat swab nucleic acid testing was negative for influenza A, influenza B, respiratory syncytial virus, adenovirus, *mycoplasma pneumoniae*, and human rhinovirus. Both blood and sputum cultures were negative. Fungal markers, including blood Aspergillus antigen (GM) and 1,3-*β*-D-glucan, were also negative. Bone marrow biopsy revealed hypercellularity with increased granulocytic and erythroid lineages, but no signs of dysplasia and no evidence of increased blasts. Chest CT (plain and contrast-enhanced) showed inflammation in the right lower lobe of the lung, a cystic mixed-density mass in the middle to upper mediastinum, located posterior to the superior vena cava and anterior to the trachea, raising suspicion for a germ cell tumor or neurogenic tumor. The lesion measured 2.9 × 3.4 × 4.1 cm, with a CT density of 19–29 HU, and showed no evidence of fat or calcification. Contrast enhancement revealed markedly uneven enhancement with patchy internal areas of enhancement (Figure 1).

**Figure 2:**
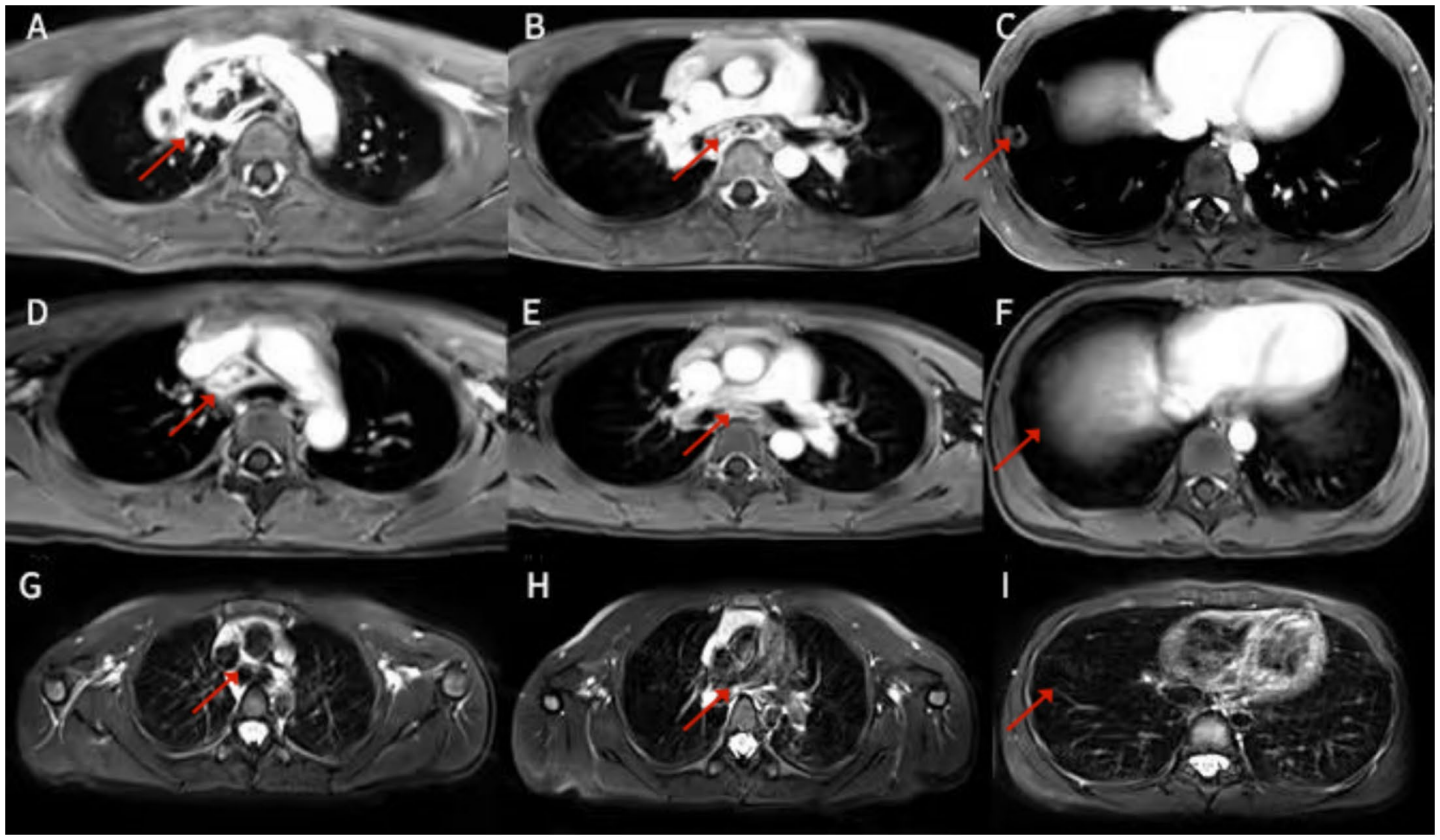
Chest MRI: Irregular abnormal signals can be seen in the upper right mediastinum, paratracheal vena cava, and right pulmonary hilum. The internal signal is uneven, mainly due to slightly longer T1 and T2 signals, and compressed fat is mainly high signal. Enhanced scanning showed significant uneven enhancement, with large areas of no enhancement visible internally **(A,B)**. After two months of comparative treatment, irregular nodule abnormal signals in the right upper mediastinum, paratracheal vena cava, and right pulmonary hilum were significantly absorbed and reduced **(D,E)**, and the mass was basically absorbed after one year of treatment **(G,H)**. Patchy/nodular slightly longer T2 signals can be seen in the lower lobe of the right lung, with higher signal intensity during lipid compression. Enhanced scanning showed uneven enhancement, with some areas showing circular enhancement **(C)** and significant absorption and reduction of patchy/nodular abnormal signals in the lower right lobe after 2 months of treatment **(F)**, as well as basic absorption after 1 year **(I)**.

**Figure 3:**
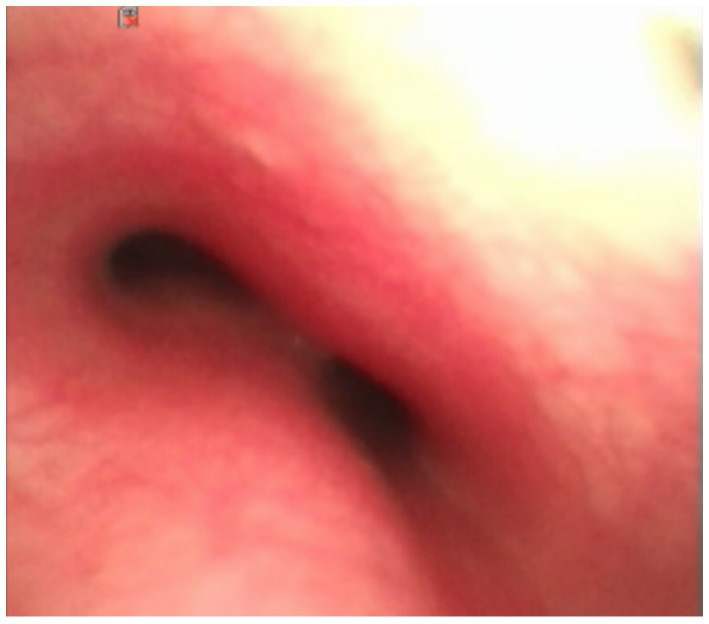
Bronchoscopy indicates external pressure on the right side of the tracheal prominence membrane.

The patient continued to experience fever on day 20 despite antibiotic therapy, prompting plans for a biopsy of the mediastinal mass. Due to the mass’s location in the middle mediastinum and its encasement of the aortic bifurcation, thoracoscopic surgery was deemed challenging, and a median sternotomy was likely necessary. Before surgery, a hospital-wide multidisciplinary team (MDT) board reviewed the case and decided to perform an additional MRI. The reason was that the chest CT plain scan and enhancement of the patient indicated lesions in the lower lobe of the right lung, right hilum, and mediastinum. In most cases, these three lesions were more likely to be explained by monism. It was difficult to distinguish infection or tumor/tumor-like lesions solely based on the CT manifestations of lesions in the right hilum and mediastinum. However, the small nodular shadow under the pleura in the lower lobe of the right lung provides valuable clues for qualitative diagnosis. After enhancement, it appeared as a circular enhancement, indicating a small abscess and suggesting that the lesion in the lower lobe of the right lung was an infectious lesion. On the other hand, multiple irregular and non-enhanced areas could be seen after enhancement of lesions in the right hilum and mediastinum, which were also lymph node distribution areas. Therefore, it was recommended to perform chest MRI plain scan and enhanced examination to further determine whether there was liquefaction necrosis area, which ultimately confirmed the presence of liquefaction necrosis in the right hilar and mediastinal lesions (Figures 2A–C). A subsequent bronchoscopy showed a normally positioned trachea with congested mucosa and suspected external compression-induced stenosis on the right side of the tracheal carina (Figure 3). The carina itself and the openings of the left and right bronchi appeared normal. No foreign bodies or granulation tissue were observed. A small amount of white secretion adhered to the bronchial mucosa in each segment, with additional white secretions floating in the bronchial lumen of various segments in the left lung. Samples were collected for bronchoalveolar lavage fluid (BALF) analysis. Fluorescent staining for fungi and acid-fast bacilli in the BALF fluid was negative.

Next, tNGS testing was preformed, and the procedures were as follows: Nucleic acids were extracted using the VAMNE Magnetic Pathogen DNA/RNA Kit (Vazyme Biotech, Nanjing, Jiangsu, China). Library construction including cDNA synthesis, target region enrichment, purification and adapter ligation, were performed using the Respiratory Pathogen Multiplex Testing Kit (King Create, Guangzhou, China). This kit targets 198 pathogens including 80 bacteria, 79 viruses (35 DNA viruses and 44 RNA viruses), 32 fungi, and 7 mycoplasmas/chlamydia Libraries were quantified using the Equability DNA HS Assay Kit (Vazyme Biotech, Nanjing, Jiangsu, China) with the Invitrogen Qubit 3.0/4.0 Fluorometer (Thermo Fisher Scientific, Waltham, MA, United States). Sequencing was performed on KM MiniseqDx-CN platform (King Create, Guangzhou, China). To ensure assay quality, Normal saline served as a negative control, *Bacillus subtilis* were used as a positive control. Data analysis was conducted using the data management and analysis system (v3.7.2, King Create). Raw sequencing reads were processed with Fastp to trim adapters and filter low-quality data Each library yielded at least 50,000 raw reads and a Q30 score >75%. High-quality reads were then aligned to a curated reference database (comprising GenBank, RefSeq, and NCBI Nucleotide entries) using Bowtie2 (v2.4.1). Taxonomic abundance was quantified as reads per 100,000 (RPhK) at the species and genus levels. Raw reads of this sample was 51,860, and Q30 score was 82.3%. After filtration, only *Cryptococcus neoformans* was detected. RPhK of *Cryptococcus neoformans* was 3,132 with amplicon coverage 100%.

Further medical history revealed that the child had been keeping two tiger-skin parrots as pets since January 2022. The child was solely responsible for feeding the birds and cleaning their cage daily, with frequent exposure to parrot feces. The parents also reported that after acquiring the parrots, the child gradually developed a poor appetite and experienced weight loss.

Since *Cryptococcus neoformans* often causes meningitis, a cerebrospinal fluid (CSF) examination was performed, including routine analysis, biochemical testing, and the three major stains for *Cryptococcus*, bacteria, and acid-fast bacilli, as well as pathogen culture. All results were negative, effectively ruling out cryptococcal meningitis. The patient was subsequently treated with a combination antifungal regimen consisting of amphotericin B and flucytosine. Follow-up chest MRI scans (plain and contrast-enhanced) were conducted at 1 and 2 months (Figures 2D–F) post-treatment. Both showed a significant reduction in the size of the mediastinal mass Additionally, the previously observed patchy and nodular shadows in the right lower lobe had markedly improved, with the nodules having completely resolved. During the course of the dual antifungal therapy, the patient developed hypokalemia due to amphotericin B, which was corrected with oral potassium supplementation. The patient also experienced elevated creatinine and blood urea nitrogen levels, along with signs of hepatic dysfunction, all attributed to amphotericin B toxicity. Consequently, the treatment regimen was adjusted to oral fluconazole for consolidation and maintenance: 12 mg/kg/day for 8 weeks followed by 6 mg/kg/day for 12 months. The most recent MRI indicated that the mediastinal mass had nearly resolved (Figures 2G–I).

## Patient perspective

As family members, looking back on the entire medical treatment process over the past year, we went through a journey from extreme anxiety to ultimate relief. At first, our child suffered from repeated high fever for 18 days and various antibiotic treatments were ineffective, leaving us feeling extremely helpless. When we learned that there was a huge mass in the child’s mediastinum and the doctor suspected it might be a germogenic tumor or a neurogenic tumor, and that a thoracotomy (midline sternotomy) might be necessary, we endured tremendous psychological pressure. We were extremely fortunate that the doctors, through discussions within MDT, decided to use non-invasive MRI examinations and BALF genetic testing (tNGS). This advanced technology not only enabled us to quickly identify the cause—a new type of cryptococcal infection—but also prevented our child from undergoing a highly traumatic surgery. After the diagnosis, we also reflected on the behavior of keeping a parrot at home and having our child clean the feces every day, realizing the importance of pet hygiene management. After a year of standardized antifungal treatment, we saw that the child’s mass almost completely disappeared and regained their former liveliness. We are very satisfied with the overall treatment plan and the long-term follow-up results, and hope to use this case to remind other bird-owning families to be aware of the potential infection risks.

## Discussion

We systematically searched PubMed, EMBASE, and Web of science and EMBASE, Scopus, and Cochrane databases for studies published between January 2010 and March 2024 using the keywords “children,” “cryptococcosis,” “mediastinal mass,” and “*Cryptococcus neoformans*.” A total of 5 eligible cases (including this case) were included, and the key clinical characteristics were compared (Table 1). All the five patients had normal immune function, and two of them had a history of bird contact. The 17-year-old case reported by Lee et al. (7) was that a lot of pigeons were raised in school, but the patient did not have direct contact with them. Our patient had a clear history of raising parrots and contact with Parrot feces, but these were found after a clear diagnosis. However, there was no history of bird contact in the cases reported by Lee et al. (8), Okachi et al. (9) and Chen et al. (10). We found that among the five patients, our case was found to be infected with *Cryptococcus neoformans* by bronchoscopy and lavage fluid ngs, and the other four cases were found by invasive lymph node biopsy (resection or acupuncture). Among them, Lee et al. (8) had a biopsy of the right supraclavicular lymph node and a biopsy of the mediastinal lymph node. Of course, in our case, because the enlargement of lymph nodes outside the mediastinum was not found, and the location was hidden, puncture biopsy could not be performed. If the median incision thoracotomy biopsy was selected, the trauma would be huge. Therefore, we successfully avoided the implementation of invasive surgery through the combination of bronchoscopy and ngs. Through literature review of these five cases, we also found cryptococcal mediastinal lesions in children with normal immunity, timely and accurate diagnosis of cryptococcal infection, standardized antifungal treatment, and generally good prognosis.

**Table 1:** Comparative table of pediatric cryptococcal mediastinal lesion cases.

Comparison dimensions	Our case	Case 2	Case 3	Case 4	Case 5
Age/Gender	7 years/Female	3 years/Female	17 years/Female	7 years/Female	17 years/Male
Immune Status	Immunocompetent	Immunocompetent	Immunocompetent	Immunocompetent	Immunocompetent
Bird Exposure History	Yes	No	Yes	No	No
Main Clinical Symptoms	Fever, anorexia, weight loss	Fever, cough, anorexia	Fever, cough, dyspnea, weight loss	Cough, fever, generalized lymphadenopathy	Cough, fever
Core Imaging Findings	Middle-upper mediastinal cystic mixed-density mass, right lower lobe inflammation with ring-enhancing nodules	Right hilar mass, confluent mediastinal lymph nodes, cavitary nodule in right lower lobe	Anterior mediastinal confluent mass (extending to right supraclavicular region), tracheal and superior vena cava compression	Mediastinal + bronchial lymphadenopathy, multiple nodular high-density shadows in both lungs, polyserosal effusion	Massive mediastinal lymphadenopathy (tracheal compression), right upper lobe consolidation with cavitation
Diagnostic Method	BALF+ tNGS	Right supraclavicular LNB + PCR + histological staining	Right supraclavicular USCB + histological staining	ILNB (Cryptococcus identified by GMS/PAS staining)	EBUS-TBNA + Grocott stain + culture + gene analysis
Induction Therapy	Amphotericin B + Flucytosine (5 weeks)	Amphotericin B → Liposomal Amphotericin B + Flucytosine (2 weeks)	Amphotericin B + Flucytosine (2 weeks)	Amphotericin B (low-dose escalation to 14 mg/d, total 6 weeks)	Liposomal Amphotericin B (intravenous, 4 weeks) + Flucytosine (oral, 2 weeks)
Consolidation + Maintenance Therapy	Fluconazole (12 mg/kg/d × 8 weeks → 6 mg/kg/d × 12 months)	Fluconazole (8 months)	Fluconazole (12 mg/kg/d × 2 months → 5 mg/kg/d × 6 months)	Voriconazole (oral)	Fluconazole (oral, total treatment duration ~5 months)
Adverse Reactions	Hypokalemia, abnormal liver and kidney function	Hypokalemia, hypotension	Hypokalemia, hypomagnesemia, nausea	Not clearly mentioned	Hypokalemia, renal dysfunction, nausea
Prognosis	Near-complete resolution of mediastinal mass after one year of treatment, clinical cure	Symptom improvement, mass reduction	Complete remission, near-complete mass absorption	Resolution of symptoms, improvement of lymphadenopathy and pulmonary nodules	Significant reduction of right upper lobe consolidation and mediastinal lymphadenopathy at 3-month follow-up, clinical recovery

EBUS-TBNA: Endobronchial ultrasound-guided transbronchial needle aspiration. BLAF: Bronchoalveolar lavage fluid; USCB: Ultrasound-guided Core Biopsy; ILNB: Inguinal lymph node biopsy; LNB: Lymph Node Biopsy.

*Cryptococcus* is the fungus that causes cryptococcosis, a fungal infection. Transmission occurs via inhalation of airborne infectious spores or desiccated yeast cells, which lodge in the alveoli, initiating pulmonary infection. This type of lung infection generally has a good prognosis, but can spread from the lungs to other parts of the body. Disseminated lesions can occur in various organs, including skin, mucous membranes, lymph nodes, bones, and internal organs. The disease may progress to involve the CNS, leading to meningitis or meningoencephalitis (4). *Cryptococcus neoformans* is responsible for approximately 95% of cryptococcal infections (11). Historically, the term “*Cryptococcus neoformans*” reflects its tendency to form tumor-like masses in the lungs and brain. Granulomatous inflammation is a hallmark of infection and plays a critical role in the containment and possible eradication of the organism (12). Immunocompromised individuals are vulnerable to infection, including those with HIV infection, other acquired immunodeficiencies caused by corticosteroids, chemotherapy, organ transplantation, or malignancy, and primary immunodeficiencies (11). However, infections can also occur in immunocompetent populations. Individuals with frequent exposure to bird droppings, such as bird caretakers, may inhale high concentrations of infectious particles (4). Once inhaled, *Cryptococcus neoformans* replicates in the lungs via budding. In some individuals, the immune system eliminates the infection; in others, the fungus remains dormant within phagocytes, reactivating if host immunity declines (13).

In children, the most common symptoms of cryptococcal disease are fever (64%), headache (55%), and vomiting (39%). Meningitis occurs in approximately 80% of cases. Pulmonary involvement is rare in HIV-positive children (1%), but significantly more common in non-HIV immunocompromised children (36%) and immunocompetent children (40%) (5). Pulmonary cryptococcosis can present as pneumonia, pleural effusion, solid lesions, hilar or mediastinal lymphadenopathy, or miliary nodules (5, 6). We speculate that the mediastinal mass in the patient is actually caused by infection of the mediastinal lymph nodes (group 2R.4R.7 lymph nodes) by *Cryptococcus neoformans*, resulting in enlargement and fusion, accompanied by infection of the hilar lymph nodes (10R) and the lower lobe of the right lung.

Bronchoscopy is a safe, minimally invasive, and well-tolerated procedure for pediatric airway assessment. BALF enables diagnostic evaluation of various pulmonary infections (14). While traditional microbial cultures remain the gold standard (15), they require long turnaround times, are low in sensitivity, and have limited capacity to detect rare or atypical pathogens (16). PCR-based detection methods are faster but rely on predesigned primers and can only identify known organisms. In contrast, NGS offers high sensitivity, cost-effectiveness, low sample volume requirements, and the ability to detect DNA and RNA pathogens simultaneously (17). In this case, NGS provided definitive evidence for *Cryptococcus neoformans* infection and prevented the need for surgical biopsy, as traditional cultures and smear microscopy were both negative.

*Cryptococcus neoformans* can also induce allergic bronchopulmonary mycosis, characterized by Th2 cytokine production, elevated IgE, eosinophilia, and macrophage activation (18). This may explain the elevated serum IgE levels observed in our patient. However, in this case, eosinophilia was not prominent.

For treatment of cryptococcal mediastinal granuloma, we followed standard protocols for severe cryptococcal infection, involving three phases: induction, consolidation, and maintenance. Induction therapy consists of intravenous liposomal amphotericin B plus flucytosine for 2–4 weeks, followed by an 8-week consolidation phase with fluconazole (19). Combination therapy significantly improves survival compared to amphotericin monotherapy (20). Maintenance therapy with low-dose fluconazole for up to one year helps prevent recurrence (21).

In conclusion, we report a rare pediatric case of mediastinal enlargement caused by cryptococcal infection through parrot exposure. Diagnosis was successfully achieved using metagenomic NGS, avoiding the trauma and risk associated with surgical biopsy.

## Data Availability

The datasets presented in this study can be found in online repositories. The names of the repository/repositories and accession number(s) can be found in the article/supplementary material.

## Glossary

CT: Computed Tomography
MRI: Magnetic Resonance Imaging
tNGS: Targeted Metagenomic Next-Generation Sequencing
BALF: Bronchoalveolar Lavage Fluid
CSF: Cerebrospinal Fluid
t-spot.tb: T-cell spot assay for tuberculosis infection
WBC: White Blood Cell
CRP: C-Reactive Protein
ESR: Erythrocyte Sedimentation Rate
GM: Galactomannan, the Aspergillus antigen
CNS: Central Nervous System
MDT: Multidisciplinary Team
IgA: Immunoglobulin A
IgE: Immunoglobulin E
IgG: Immunoglobulin G
IgM: Immunoglobulin M
C3: Complement 3
C4: Complement 4
IFN-*γ*: Interferon-Gamma
IL-2: Interleukin-2
IL-4: Interleukin-4
IL-6: Interleukin-6
IL-10: Interleukin-10
IL-17: Interleukin-17
TNF-*α*: Tumor Necrosis Factor-Alpha
EBV: Epstein–Barr Virus
RSV: Respiratory Syncytial Virus
T-ALL: T-Cell Acute Lymphoblastic Leukemia
AFP: Alpha-Fetoprotein
CEA: Carcinoembryonic Antigen
